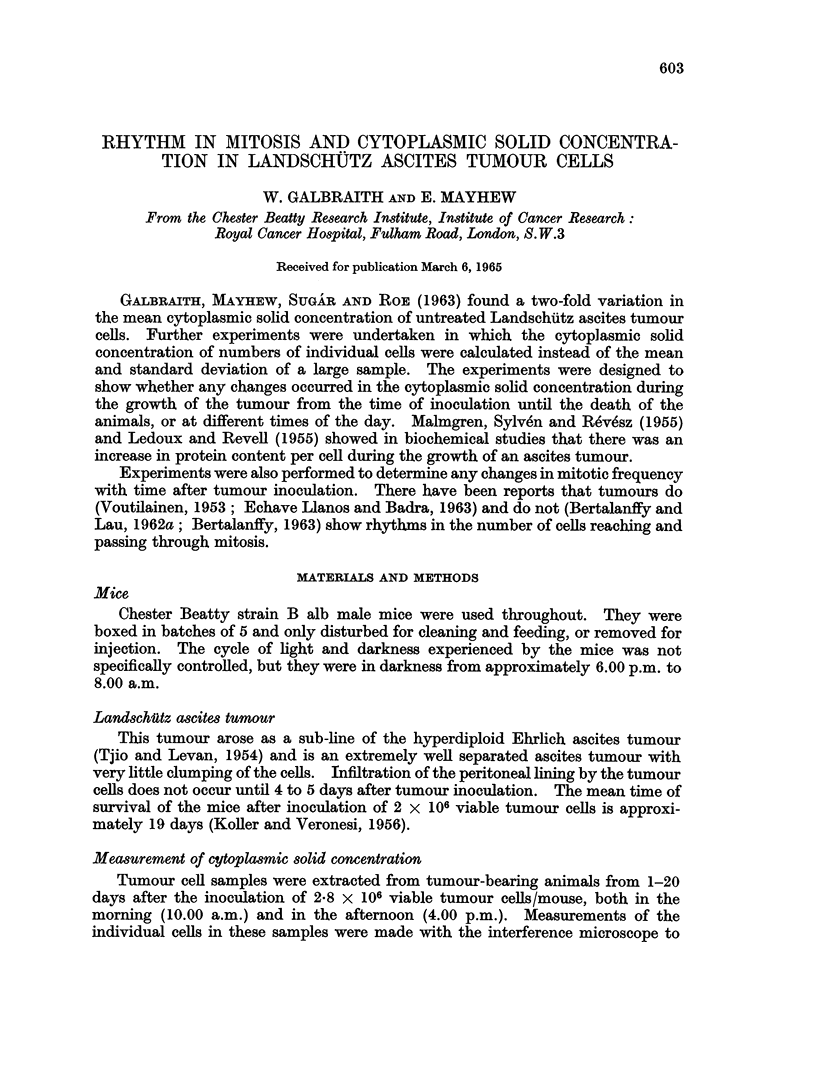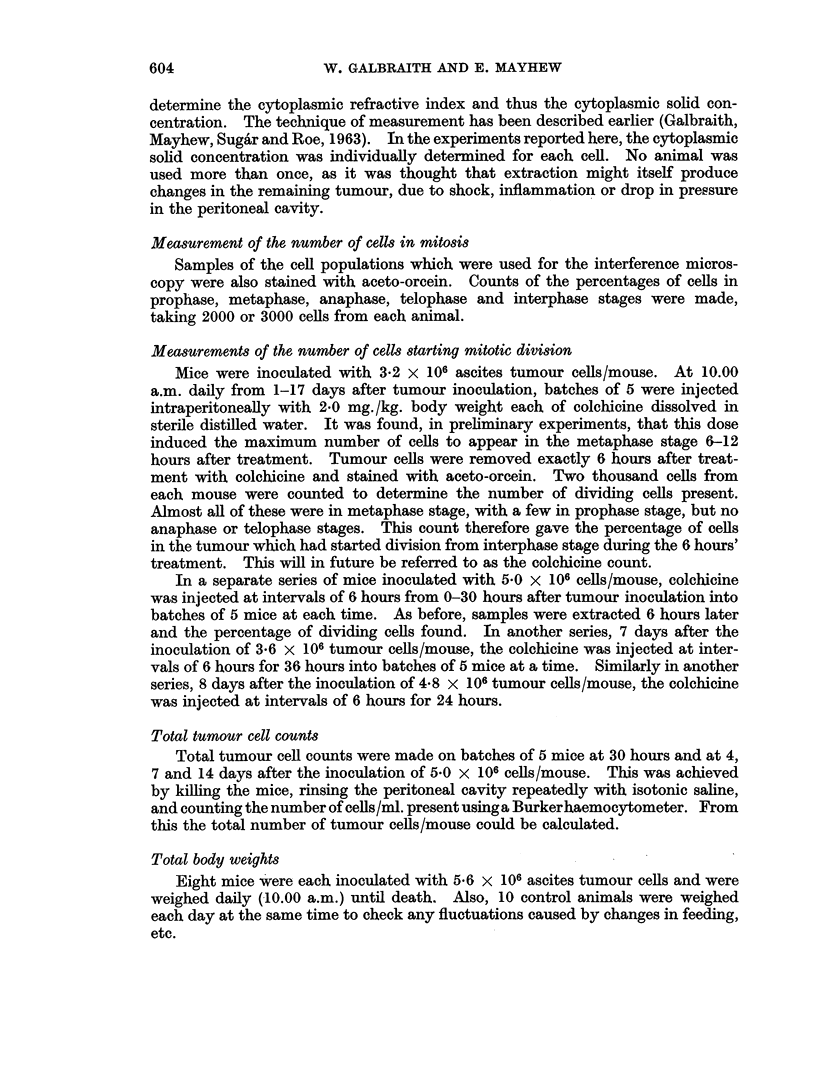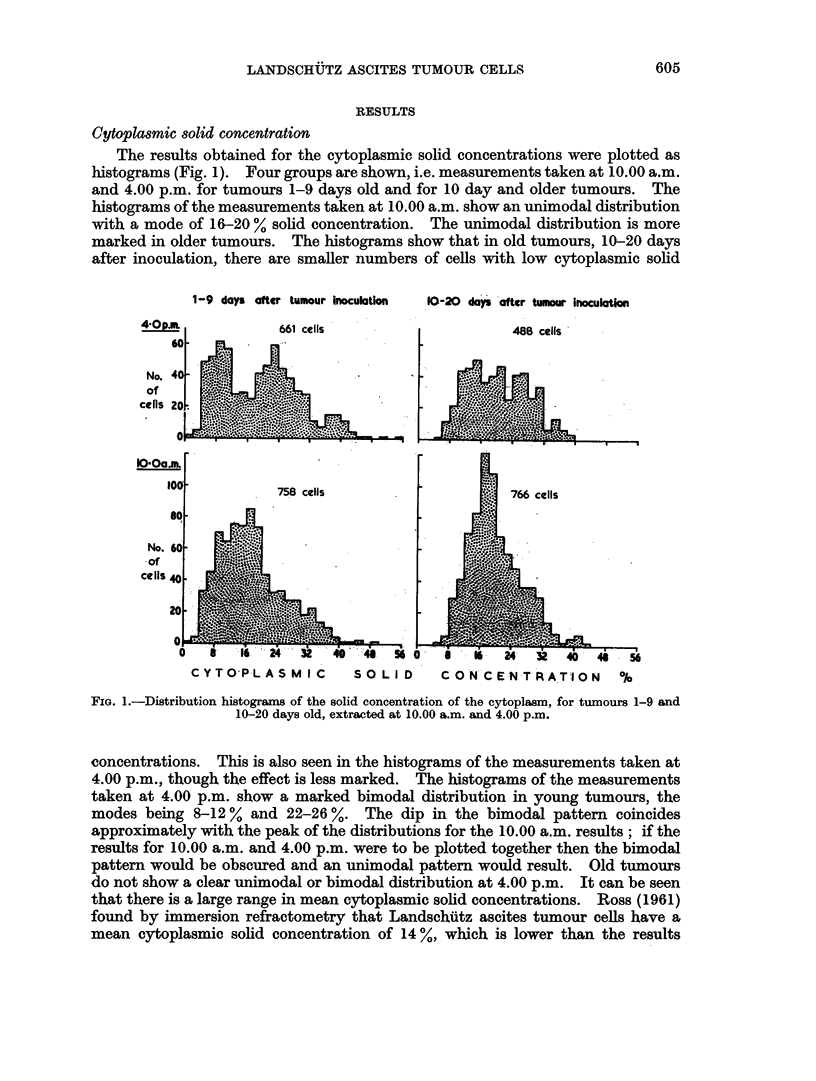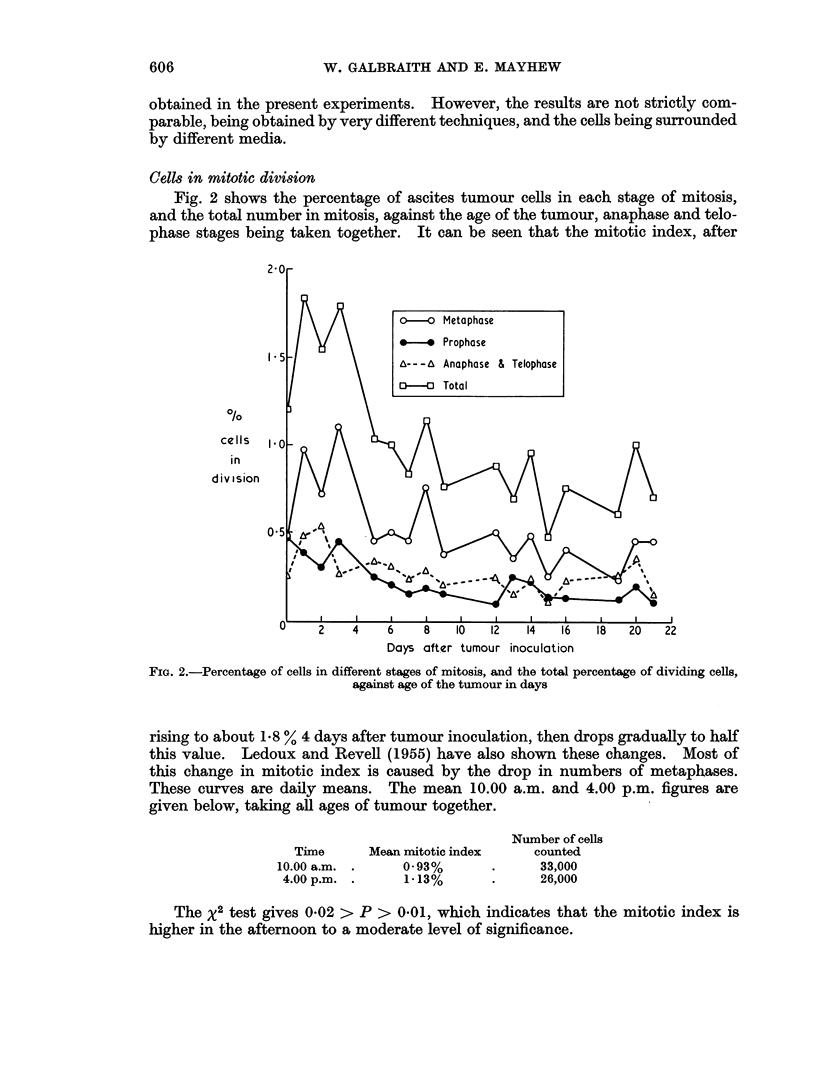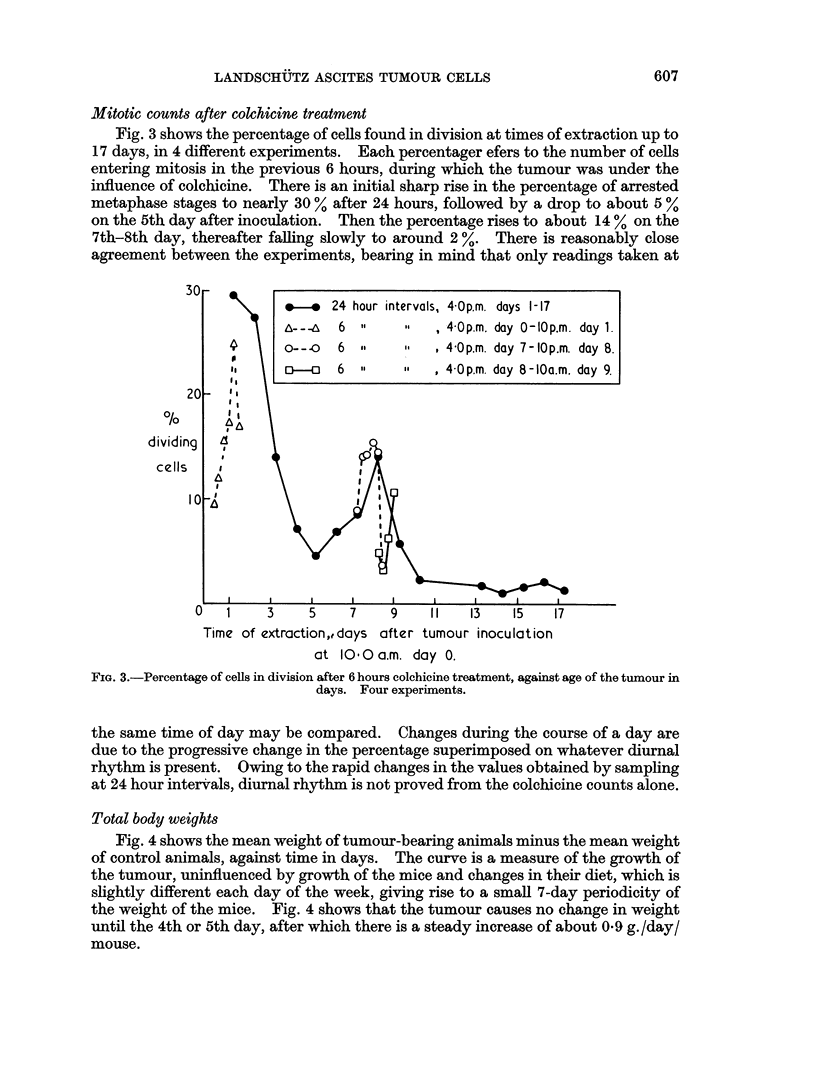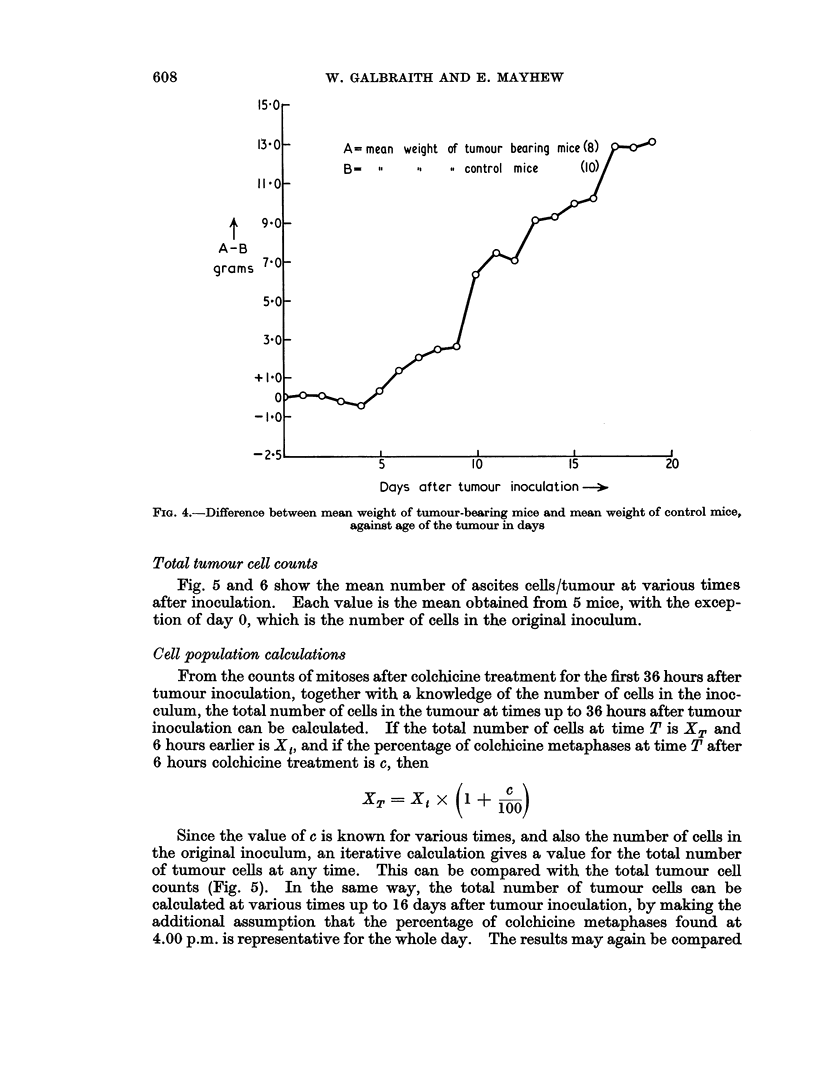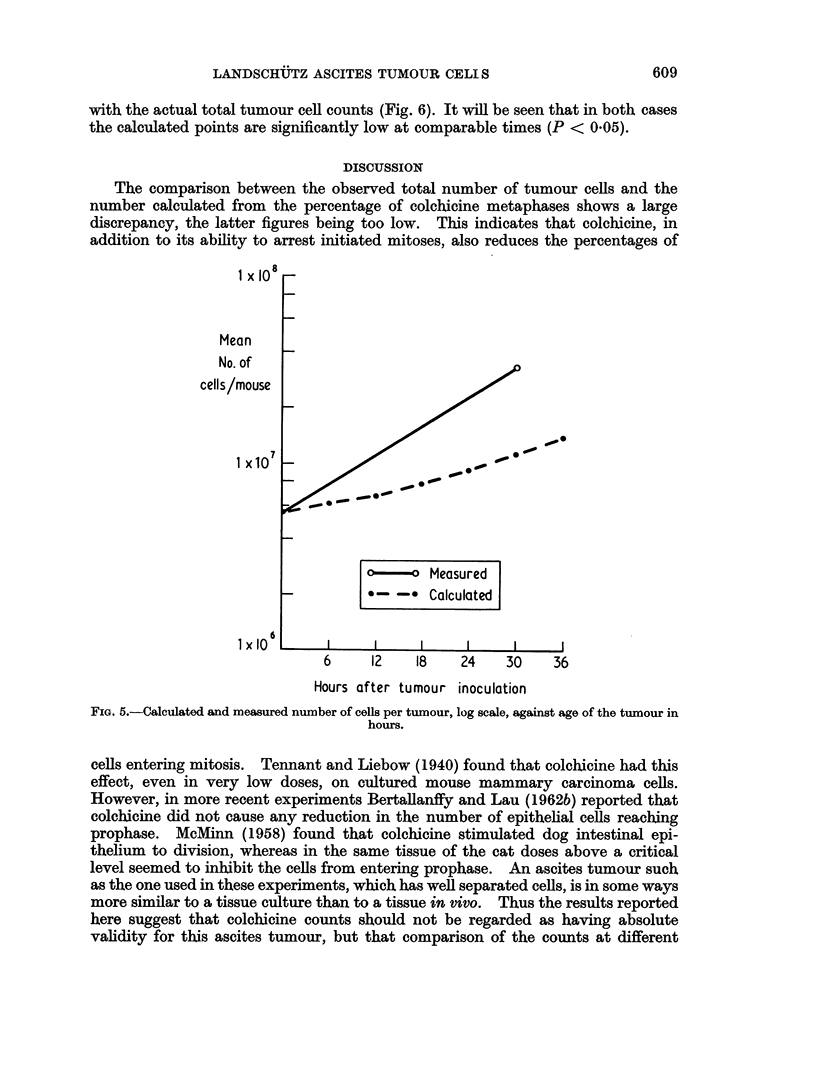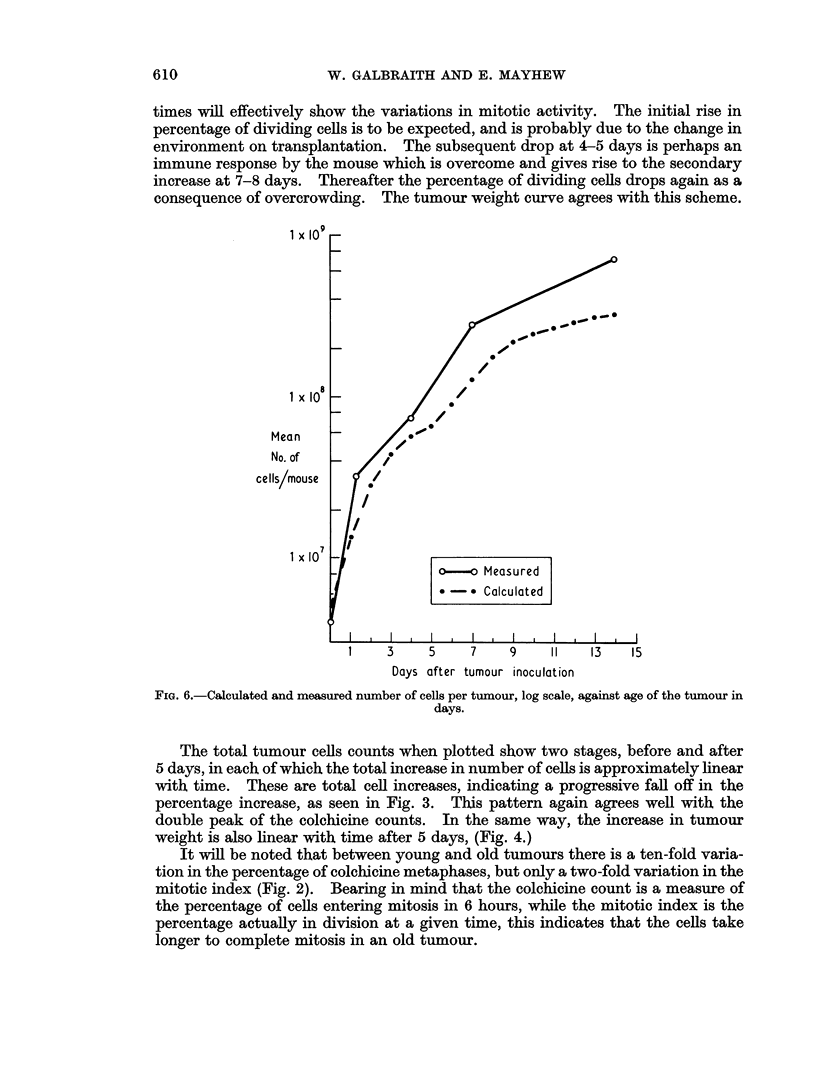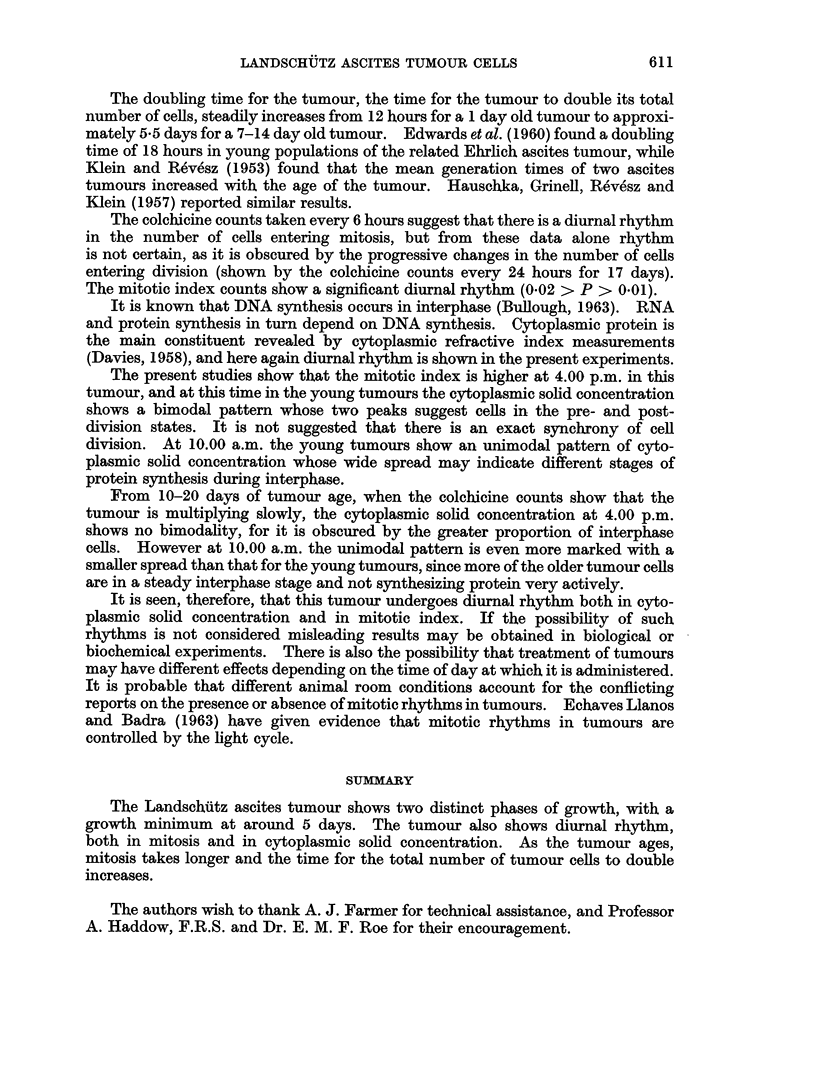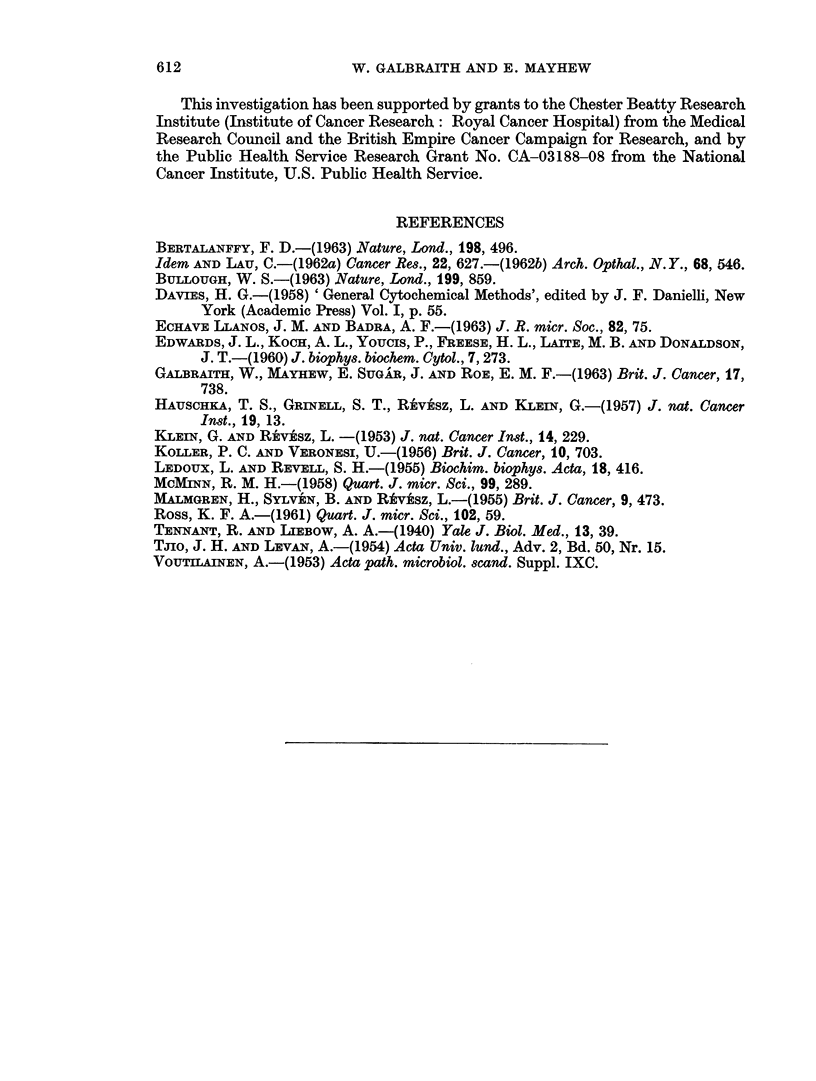# Rhythm in mitosis and cytoplasmic solid concentration in Landschütz ascites tumour cells.

**DOI:** 10.1038/bjc.1965.76

**Published:** 1965-09

**Authors:** W. Galbraith, E. Mayhew


### RHYTHM IN MITOSIS AND CYTOPLASMIC SOLID CONCENTRATION IN LANDSCHÜTZ ASCITES TUMOUR CELLS

W. GALBRAITH AND E. MAYHEW

*From the Chester Beatty Research Institute, Institute of Cancer Research : Royal Cancer Hospital, Fulham Road, London, S.W.3*

Received for publication March 6, 1965

GALBRAITH, MAYHEW, SUGÁR AND ROE (1963) found a two-fold variation in the mean cytoplasmic solid concentration of untreated Landschütz ascites tumour cells. Further experiments were undertaken in which the cytoplasmic solid concentration of numbers of individual cells were calculated instead of the mean and standard deviation of a large sample. The experiments were designed to show whether any changes occurred in the cytoplasmic solid concentration during the growth of the tumour from the time of inoculation until the death of the animals, or at different times of the day. Malmgren, Sylvén and Révész (1955) and Ledoux and Revell (1955) showed in biochemical studies that there was an increase in protein content per cell during the growth of an ascites tumour.

Experiments were also performed to determine any changes in mitotic frequency with time after tumour inoculation. There have been reports that tumours do (Voutilainen, 1953 ; Echave Llanos and Badra, 1963) and do not (Bertalanffy and Lau, 1962*a* ; Bertalanffy, 1963) show rhythms in the number of cells reaching and passing through mitosis.

### MATERIALS AND METHODS

### *Mice*

Chester Beatty strain B alb male mice were used throughout. They were boxed in batches of 5 and only disturbed for cleaning and feeding, or removed for injection. The cycle of light and darkness experienced by the mice was not specifically controlled, but they were in darkness from approximately 6.00 p.m. to 8.00 a.m.

### *Landschütz ascites tumour*

This tumour arose as a sub-line of the hyperdiploid Ehrlich ascites tumour (Tjio and Levan, 1954) and is an extremely well separated ascites tumour with very little clumping of the cells. Infiltration of the peritoneal lining by the tumour cells does not occur until 4 to 5 days after tumour inoculation. The mean time of survival of the mice after inoculation of $2 \times 10^6$ viable tumour cells is approximately 19 days (Koller and Veronesi, 1956).

### *Measurement of cytoplasmic solid concentration*

Tumour cell samples were extracted from tumour-bearing animals from 1–20 days after the inoculation of $2{\cdot}8 \times 10^6$ viable tumour cells/mouse, both in the morning (10.00 a.m.) and in the afternoon (4.00 p.m.). Measurements of the individual cells in these samples were made with the interference microscope to determine the cytoplasmic refractive index and thus the cytoplasmic solid concentration. The technique of measurement has been described earlier (Galbraith, Mayhew, Sugár and Roe, 1963). In the experiments reported here, the cytoplasmic solid concentration was individually determined for each cell. No animal was used more than once, as it was thought that extraction might itself produce changes in the remaining tumour, due to shock, inflammation or drop in pressure in the peritoneal cavity.

*Measurement of the number of cells in mitosis*

Samples of the cell populations which were used for the interference microscopy were also stained with aceto-orcein. Counts of the percentages of cells in prophase, metaphase, anaphase, telophase and interphase stages were made, taking 2000 or 3000 cells from each animal.

*Measurements of the number of cells starting mitotic division*

Mice were inoculated with $3{\cdot}2 \times 10^6$ ascites tumour cells/mouse. At 10.00 a.m. daily from 1–17 days after tumour inoculation, batches of 5 were injected intraperitoneally with 2·0 mg./kg. body weight each of colchicine dissolved in sterile distilled water. It was found, in preliminary experiments, that this dose induced the maximum number of cells to appear in the metaphase stage 6–12 hours after treatment. Tumour cells were removed exactly 6 hours after treatment with colchicine and stained with aceto-orcein. Two thousand cells from each mouse were counted to determine the number of dividing cells present. Almost all of these were in metaphase stage, with a few in prophase stage, but no anaphase or telophase stages. This count therefore gave the percentage of cells in the tumour which had started division from interphase stage during the 6 hours' treatment. This will in future be referred to as the colchicine count.

In a separate series of mice inoculated with $5{\cdot}0 \times 10^6$ cells/mouse, colchicine was injected at intervals of 6 hours from 0–30 hours after tumour inoculation into batches of 5 mice at each time. As before, samples were extracted 6 hours later and the percentage of dividing cells found. In another series, 7 days after the inoculation of $3{\cdot}6 \times 10^6$ tumour cells/mouse, the colchicine was injected at intervals of 6 hours for 36 hours into batches of 5 mice at a time. Similarly in another series, 8 days after the inoculation of $4{\cdot}8 \times 10^6$ tumour cells/mouse, the colchicine was injected at intervals of 6 hours for 24 hours.

*Total tumour cell counts*

Total tumour cell counts were made on batches of 5 mice at 30 hours and at 4, 7 and 14 days after the inoculation of $5{\cdot}0 \times 10^6$ cells/mouse. This was achieved by killing the mice, rinsing the peritoneal cavity repeatedly with isotonic saline, and counting the number of cells/ml. present using a Burker haemocytometer. From this the total number of tumour cells/mouse could be calculated.

*Total body weights*

Eight mice were each inoculated with $5{\cdot}6 \times 10^6$ ascites tumour cells and were weighed daily (10.00 a.m.) until death. Also, 10 control animals were weighed each day at the same time to check any fluctuations caused by changes in feeding, etc.

### RESULTS

### *Cytoplasmic solid concentration*

The results obtained for the cytoplasmic solid concentrations were plotted as histograms (Fig. 1). Four groups are shown, i.e. measurements taken at 10.00 a.m. and 4.00 p.m. for tumours 1–9 days old and for 10 day and older tumours. The histograms of the measurements taken at 10.00 a.m. show an unimodal distribution with a mode of 16–20 % solid concentration. The unimodal distribution is more marked in older tumours. The histograms show that in old tumours, 10–20 days after inoculation, there are smaller numbers of cells with low cytoplasmic solid

FIG. 1.—Distribution histograms of the solid concentration of the cytoplasm, for tumours 1–9 and 10–20 days old, extracted at 10.00 a.m. and 4.00 p.m.

concentrations. This is also seen in the histograms of the measurements taken at 4.00 p.m., though the effect is less marked. The histograms of the measurements taken at 4.00 p.m. show a marked bimodal distribution in young tumours, the modes being 8–12 % and 22–26 %. The dip in the bimodal pattern coincides approximately with the peak of the distributions for the 10.00 a.m. results ; if the results for 10.00 a.m. and 4.00 p.m. were to be plotted together then the bimodal pattern would be obscured and an unimodal pattern would result. Old tumours do not show a clear unimodal or bimodal distribution at 4.00 p.m. It can be seen that there is a large range in mean cytoplasmic solid concentrations. Ross (1961) found by immersion refractometry that Landschütz ascites tumour cells have a mean cytoplasmic solid concentration of 14 %, which is lower than the results obtained in the present experiments. However, the results are not strictly comparable, being obtained by very different techniques, and the cells being surrounded by different media.

*Cells in mitotic division*

Fig. 2 shows the percentage of ascites tumour cells in each stage of mitosis, and the total number in mitosis, against the age of the tumour, anaphase and telophase stages being taken together. It can be seen that the mitotic index, after

FIG. 2.—Percentage of cells in different stages of mitosis, and the total percentage of dividing cells, against age of the tumour in days rising to about 1·8 % 4 days after tumour inoculation, then drops gradually to half this value. Ledoux and Revell (1955) have also shown these changes. Most of this change in mitotic index is caused by the drop in numbers of metaphases. These curves are daily means. The mean 10.00 a.m. and 4.00 p.m. figures are given below, taking all ages of tumour together.

| Time | Mean mitotic index | Number of cells counted |
|---|---|---|
| 10.00 a.m. . | 0·93% . | 33,000 |
| 4.00 p.m. . | 1·13% . | 26,000 |

The $\chi^2$ test gives $0{\cdot}02 > P > 0{\cdot}01$, which indicates that the mitotic index is higher in the afternoon to a moderate level of significance.

*Mitotic counts after colchicine treatment*

Fig. 3 shows the percentage of cells found in division at times of extraction up to 17 days, in 4 different experiments. Each percentager efers to the number of cells entering mitosis in the previous 6 hours, during which the tumour was under the influence of colchicine. There is an initial sharp rise in the percentage of arrested metaphase stages to nearly 30 % after 24 hours, followed by a drop to about 5 % on the 5th day after inoculation. Then the percentage rises to about 14 % on the 7th–8th day, thereafter falling slowly to around 2 %. There is reasonably close agreement between the experiments, bearing in mind that only readings taken at

Fig. 3.—Percentage of cells in division after 6 hours colchicine treatment, against age of the tumour in days. Four experiments.

the same time of day may be compared. Changes during the course of a day are due to the progressive change in the percentage superimposed on whatever diurnal rhythm is present. Owing to the rapid changes in the values obtained by sampling at 24 hour intervals, diurnal rhythm is not proved from the colchicine counts alone.

*Total body weights*

Fig. 4 shows the mean weight of tumour-bearing animals minus the mean weight of control animals, against time in days. The curve is a measure of the growth of the tumour, uninfluenced by growth of the mice and changes in their diet, which is slightly different each day of the week, giving rise to a small 7-day periodicity of the weight of the mice. Fig. 4 shows that the tumour causes no change in weight until the 4th or 5th day, after which there is a steady increase of about 0·9 g./day/mouse.

FIG. 4.—Difference between mean weight of tumour-bearing mice and mean weight of control mice, against age of the tumour in days

*Total tumour cell counts*

Fig. 5 and 6 show the mean number of ascites cells/tumour at various times after inoculation. Each value is the mean obtained from 5 mice, with the exception of day 0, which is the number of cells in the original inoculum.

*Cell population calculations*

From the counts of mitoses after colchicine treatment for the first 36 hours after tumour inoculation, together with a knowledge of the number of cells in the inoculum, the total number of cells in the tumour at times up to 36 hours after tumour inoculation can be calculated. If the total number of cells at time $T$ is $X_T$ and 6 hours earlier is $X_t$, and if the percentage of colchicine metaphases at time $T$ after 6 hours colchicine treatment is $c$, then

$$X_T = X_t \times \left(1 + \frac{c}{100}\right)$$

Since the value of $c$ is known for various times, and also the number of cells in the original inoculum, an iterative calculation gives a value for the total number of tumour cells at any time. This can be compared with the total tumour cell counts (Fig. 5). In the same way, the total number of tumour cells can be calculated at various times up to 16 days after tumour inoculation, by making the additional assumption that the percentage of colchicine metaphases found at 4.00 p.m. is representative for the whole day. The results may again be compared with the actual total tumour cell counts (Fig. 6). It will be seen that in both cases the calculated points are significantly low at comparable times ($P < 0{\cdot}05$).

### DISCUSSION

The comparison between the observed total number of tumour cells and the number calculated from the percentage of colchicine metaphases shows a large discrepancy, the latter figures being too low. This indicates that colchicine, in addition to its ability to arrest initiated mitoses, also reduces the percentages of

FIG. 5.—Calculated and measured number of cells per tumour, log scale, against age of the tumour in hours.

cells entering mitosis. Tennant and Liebow (1940) found that colchicine had this effect, even in very low doses, on cultured mouse mammary carcinoma cells. However, in more recent experiments Bertallanffy and Lau (1962*b*) reported that colchicine did not cause any reduction in the number of epithelial cells reaching prophase. McMinn (1958) found that colchicine stimulated dog intestinal epithelium to division, whereas in the same tissue of the cat doses above a critical level seemed to inhibit the cells from entering prophase. An ascites tumour such as the one used in these experiments, which has well separated cells, is in some ways more similar to a tissue culture than to a tissue *in vivo*. Thus the results reported here suggest that colchicine counts should not be regarded as having absolute validity for this ascites tumour, but that comparison of the counts at different times will effectively show the variations in mitotic activity. The initial rise in percentage of dividing cells is to be expected, and is probably due to the change in environment on transplantation. The subsequent drop at 4–5 days is perhaps an immune response by the mouse which is overcome and gives rise to the secondary increase at 7–8 days. Thereafter the percentage of dividing cells drops again as a consequence of overcrowding. The tumour weight curve agrees with this scheme.

FIG. 6.—Calculated and measured number of cells per tumour, log scale, against age of the tumour in days.

The total tumour cells counts when plotted show two stages, before and after 5 days, in each of which the total increase in number of cells is approximately linear with time. These are total cell increases, indicating a progressive fall off in the percentage increase, as seen in Fig. 3. This pattern again agrees well with the double peak of the colchicine counts. In the same way, the increase in tumour weight is also linear with time after 5 days, (Fig. 4.)

It will be noted that between young and old tumours there is a ten-fold variation in the percentage of colchicine metaphases, but only a two-fold variation in the mitotic index (Fig. 2). Bearing in mind that the colchicine count is a measure of the percentage of cells entering mitosis in 6 hours, while the mitotic index is the percentage actually in division at a given time, this indicates that the cells take longer to complete mitosis in an old tumour.

The doubling time for the tumour, the time for the tumour to double its total number of cells, steadily increases from 12 hours for a 1 day old tumour to approximately 5·5 days for a 7–14 day old tumour. Edwards *et al.* (1960) found a doubling time of 18 hours in young populations of the related Ehrlich ascites tumour, while Klein and Révész (1953) found that the mean generation times of two ascites tumours increased with the age of the tumour. Hauschka, Grinell, Révész and Klein (1957) reported similar results.

The colchicine counts taken every 6 hours suggest that there is a diurnal rhythm in the number of cells entering mitosis, but from these data alone rhythm is not certain, as it is obscured by the progressive changes in the number of cells entering division (shown by the colchicine counts every 24 hours for 17 days). The mitotic index counts show a significant diurnal rhythm ($0{\cdot}02 > P > 0{\cdot}01$).

It is known that DNA synthesis occurs in interphase (Bullough, 1963). RNA and protein synthesis in turn depend on DNA synthesis. Cytoplasmic protein is the main constituent revealed by cytoplasmic refractive index measurements (Davies, 1958), and here again diurnal rhythm is shown in the present experiments.

The present studies show that the mitotic index is higher at 4.00 p.m. in this tumour, and at this time in the young tumours the cytoplasmic solid concentration shows a bimodal pattern whose two peaks suggest cells in the pre- and post-division states. It is not suggested that there is an exact synchrony of cell division. At 10.00 a.m. the young tumours show an unimodal pattern of cytoplasmic solid concentration whose wide spread may indicate different stages of protein synthesis during interphase.

From 10–20 days of tumour age, when the colchicine counts show that the tumour is multiplying slowly, the cytoplasmic solid concentration at 4.00 p.m. shows no bimodality, for it is obscured by the greater proportion of interphase cells. However at 10.00 a.m. the unimodal pattern is even more marked with a smaller spread than that for the young tumours, since more of the older tumour cells are in a steady interphase stage and not synthesizing protein very actively.

It is seen, therefore, that this tumour undergoes diurnal rhythm both in cytoplasmic solid concentration and in mitotic index. If the possibility of such rhythms is not considered misleading results may be obtained in biological or biochemical experiments. There is also the possibility that treatment of tumours may have different effects depending on the time of day at which it is administered. It is probable that different animal room conditions account for the conflicting reports on the presence or absence of mitotic rhythms in tumours. Echaves Llanos and Badra (1963) have given evidence that mitotic rhythms in tumours are controlled by the light cycle.

### SUMMARY

The Landschütz ascites tumour shows two distinct phases of growth, with a growth minimum at around 5 days. The tumour also shows diurnal rhythm, both in mitosis and in cytoplasmic solid concentration. As the tumour ages, mitosis takes longer and the time for the total number of tumour cells to double increases.

The authors wish to thank A. J. Farmer for technical assistance, and Professor A. Haddow, F.R.S. and Dr. E. M. F. Roe for their encouragement.

This investigation has been supported by grants to the Chester Beatty Research Institute (Institute of Cancer Research : Royal Cancer Hospital) from the Medical Research Council and the British Empire Cancer Campaign for Research, and by the Public Health Service Research Grant No. CA–03188–08 from the National Cancer Institute, U.S. Public Health Service.

### REFERENCES

BERTALANFFY, F. D.—(1963) *Nature, Lond.*, **198**, 496.

*Idem* AND LAU, C.—(1962*a*) *Cancer Res.*, **22**, 627.—(1962*b*) *Arch. Opthal., N.Y.*, **68**, 546.

BULLOUGH, W. S.—(1963) *Nature, Lond.*, **199**, 859.

DAVIES, H. G.—(1958) ' General Cytochemical Methods', edited by J. F. Danielli, New York (Academic Press) Vol. I, p. 55.

ECHAVE LLANOS, J. M. AND BADRA, A. F.—(1963) *J. R. micr. Soc.*, **82**, 75.

EDWARDS, J. L., KOCH, A. L., YOUCIS, P., FREESE, H. L., LAITE, M. B. AND DONALDSON, J. T.—(1960) *J. biophys. biochem. Cytol.*, **7**, 273.

GALBRAITH, W., MAYHEW, E. SUGÁR, J. AND ROE, E. M. F.—(1963) *Brit. J. Cancer*, **17**, 738.

HAUSCHKA, T. S., GRINELL, S. T., RÉVÉSZ, L. AND KLEIN, G.—(1957) *J. nat. Cancer Inst.*, **19**, 13.

KLEIN, G. AND RÉVÉSZ, L. —(1953) *J. nat. Cancer Inst.*, **14**, 229.

KOLLER, P. C. AND VERONESI, U.—(1956) *Brit. J. Cancer*, **10**, 703.

LEDOUX, L. AND REVELL, S. H.—(1955) *Biochim. biophys. Acta*, **18**, 416.

MCMINN, R. M. H.—(1958) *Quart. J. micr. Sci.*, **99**, 289.

MALMGREN, H., SYLVÉN, B. AND RÉVÉSZ, L.—(1955) *Brit. J. Cancer*, **9**, 473.

ROSS, K. F. A.—(1961) *Quart. J. micr. Sci.*, **102**, 59.

TENNANT, R. AND LIEBOW, A. A.—(1940) *Yale J. Biol. Med.*, **13**, 39.

TJIO, J. H. AND LEVAN, A.—(1954) *Acta Univ. lund.*, Adv. 2, Bd. 50, Nr. 15.

VOUTILAINEN, A.—(1953) *Acta path. microbiol. scand.* Suppl. IXC.